# Brachial Plexus Anatomy of Sprague Dawley Rat Compared to Human

**DOI:** 10.1055/a-2591-2757

**Published:** 2025-05-29

**Authors:** Alison N. Jacobs, Luke J. Bolstad, Natalie Martinson, Ethan Mickelson, Matthew R. Ceelen, Owen R. Lefebvre, Roy Ram Klein, Daniel J. Hellenbrand, Amgad S. Hanna

**Affiliations:** 1 Department of Neurological Surgery, University of Wisconsin School of Medicine and Public Health (UWSMPH), Madison, Wisconsin, United States; 2 Department of Biomedical Engineering, University of Wisconsin Madison, Madison, Wisconsin, United States

**Keywords:** anatomy, avulsion, brachial plexus, nerve injury, Sprague Dawley Rat

## Abstract

Brachial plexus injury (BPI) occurs when the brachial plexus (BP) is compressed, stretched, or avulsed. A mild BPI results in acute arm pain, tingling, or numbness, while more severe injuries can lead to permanent muscle weakness or loss of function of the extremity if left untreated. Many BPI treatments developed in small animal models fail to translate effectively to human clinical trials. Furthermore, there is a lack of comparative studies exploring the anatomical differences between BPs in different species. The objective of this study is to compare the BP anatomy between humans and Sprague–Dawley (SD) rats to determine if the SD rat is a suitable model for studying BPI mechanisms and treatments. Four human BPs were compared to five SD rat BPs. Gross anatomical analysis revealed mild similarities in the branching patterns of SD rat and human BP. Histological results indicated that SD rats had significantly smaller musculocutaneous ( *p* = 0.0095), median ( *p* < 0.0001), and ulnar ( *p* < 0.0001) nerves compared to humans. Additionally, SD rats had significantly fewer axons than humans in the musculocutaneous ( *p* = 0.0190), median ( *p* < 0.0001), and ulnar nerves ( *p* < 0.0001). Due to the anatomical and histological differences between the two species, therapeutic interventions for BPIs developed in rats should be further tested in a larger animal model, such as the Wisconsin Miniature Swine, before progressing to human clinical trials.

## Introduction

The brachial plexus (BP) is a complex network of nerves (C5-T1) responsible for innervating muscles of the upper extremities. A brachial plexus injury (BPI) occurs when one or more of these nerves are stretched, compressed, or avulsed. The prognosis for BPI varies based on the severity of the injury. 1 Mild BPIs, such as stingers and burners, cause temporary pain in the upper arm, while severe injuries, such as avulsions and pan-plexus palsy, can lead to permanent muscle weakness or loss of limb function if left untreated. 2 3

BPIs affect approximately 1% of multi-trauma victims, predominantly as a result of traffic accidents. 4 The average age of individuals suffering from BPI ranges from 25 to 29 years old. These injuries impose significant socioeconomic and psychological stress on these young individuals, with the median long-term indirect cost of $801,723 per case. 5 6

A BPI is challenging to treat due to the complex nature of the injury and the lack of diversity in specific care options. 7 Current treatment methods include nerve grafts, nerve transfers, free muscle flaps, and tendon transfers. 8 While these interventions have been shown to restore some motor function of the upper extremity and help limit the degree of pain experienced by patients, they often fail to restore sensory function. 9 10 Despite recent advancements in nerve repair techniques, the prognosis of BPI remains poor. To address this challenge and develop better treatments, there is a need for an animal model that can recapitulate the anatomy and pathophysiology associated with human BPI. 11

Small animal models, such as rats, mice, and rabbits, offer consistent replicable genetics and low husbandry maintenance. However, treatment methods developed in these models often fail to produce satisfactory results in human clinical trials. Differences in the size of the BP, physiological response, and anatomical orientation of the nerves contribute to the failure of treatments translating to human clinical trials. 11 12 13 These limitations should be considered when developing experimental treatments for BPI. Currently, there is limited research investigating the nerve anatomy of the rat BP. 14 Additionally, there is a lack of sufficient in vivo research methods to meet the clinical needs for repairing larger nerve gaps in humans. 15 Therefore, it is essential to explore models that can bridge the gap between small animals and human clinical trials.

This study aims to compare the anatomy of the BP in humans and Sprague–Dawley (SD) rats to determine whether rats serve as a suitable model for studying BPI. Dissections were performed to analyze and compare the gross anatomy of the BP between species, while their microanatomy was compared through histological analyses.

## Methods

### Species

The human gross anatomy and microanatomy results from the previous study by Hanna et al were used in the current study. 16 The specimens, derived from three males and one female were dissected and analyzed to explore the anatomical and physiological aspects of the BP. ( Table 1 ). To compare to the human BP anatomy, five SD rats, 10 to 14 weeks old, were dissected. The specimens consisted of three males and two females. A comparative analysis of the median, ulnar, musculocutaneous, radial, axillary, and suprascapular nerves was performed between the two species. All experiments involving animals were conducted under protocols approved by the University of Wisconsin–Madison Institutional Animal Care and Use Committee in accordance with published NIH and USDA guidelines.

**Table 1:** Histological specimen details

Specimen	Age	Samples	Underlying condition
Human male	57 y	Median, ulnar, and musculocutaneous	Patient had hepatitis C
Human female	61 y	Median, ulnar, and musculocutaneous	Patient had neuropathy
Human male	34 y	Median, ulnar, and musculocutaneous	None
Human male	60 y	Radial, axillary, and suprascapular	Patient was obese
SD rat male	102 d	Median, ulnar, musculocutaneous, radial, axillary, and suprascapular	None
SD rat female	101 d	Median, ulnar, musculocutaneous, radial, axillary, and suprascapular	None

### Rat Dissections

Five SD rats (weight = 278.5 ± 17.99 g; age = 101.25 ± 11.44 days; mean ± SEM) were euthanized to examine the gross anatomy of seven BPs (bilateral in two rats). Euthanasia began by sedating rats with isoflurane, followed by an intracardiac perfusion using 0.9% saline solution. To expose the BP, an anterior dissection was performed. The dissection began with an incision from one elbow to the other, followed by a second incision at the deltopectoral groove. The deltoid and pectoral muscles were then resected, and the clavicle was removed. The forelimb was abducted to ensure full exposure to the BP.

### Histological Analysis

Of the rats utilized, one male and one female (weight = 309 and 255 g; age = 102 and 101 days) were further dissected to examine the histology of the rat BP. A total of four BPs were harvested (two from each rat) for the following nerve samples: median, ulnar, musculocutaneous, radial, axillary, and suprascapular. For comparison, the same nerves were obtained from four fresh human cadavers. To ensure consistent analysis, both rat and human samples underwent the same processing and fixation procedures, as postprocessing steps can cause nerve tissues to shrink. 17 All nerve segments were submerged in 0.1 M PBS, containing 2.5% glutaraldehyde, for a minimum of 24 hours. To view the myelinated axons, the entire nerve samples were rinsed twice in 1× PBS, placed in 2% osmium tetroxide in 1× PBS for 2 hours, dehydrated in ethanol, and paraffin-embedded. 18 The paraffin-embedded segments were then sectioned transversely 5 µm thick, with four sections placed on each glass slide. Tissue sections were counter-stained with Masson's Trichrome (Sigma) and the cover slipped with Permount. Two sections from each nerve segment were left without counterstaining, retaining only the osmium tetroxide fixation, to facilitate the counting of myelinated axons.

All sections were captured using the Keyence BZ-9000 microscope. The myelinated axons were assessed using Keyence BZ-II Analyzer software. The axons were filled, and the total area of each myelinated axon was recorded. Only myelinated axons larger than 3 μm ^2^ were analyzed and counted. The cross-sectional area of the nerve trunk was measured by outlining the epineurium using ImageJ.

### Statistical Analysis

A two-tailed, two-sample *t* -test was used to compare nerve size and axon count between the two species using GraphPad Prism 10. To compare axon size between the two species, an estimated geometric mean was calculated after exponentiating estimates from log-transformed data analyzed via mixed-effects ANOVA with the specimen as a random effect. Quantitative nerve size, axon count, and axon size data in the text and figures are reported as mean (95% CI). Differences were considered significant at *p* < 0.05

## Results

### Human BP Gross Anatomy

**Fig. 1:**
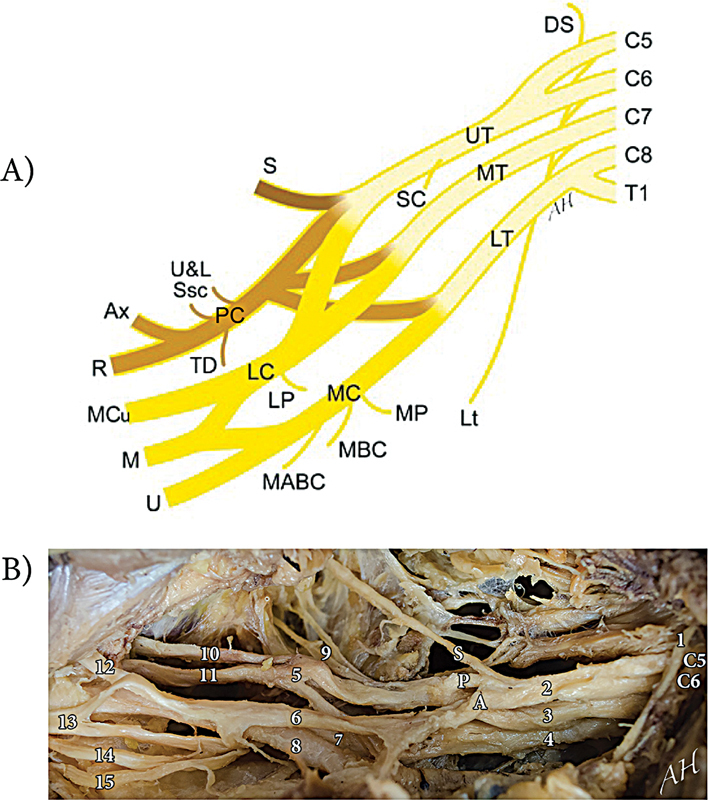
Human brachial plexus. ( **A** ) Diagram of the human brachial plexus. ( **B** ) Anterior exposure of the human right brachial plexus with the clavicle removed. Note the SPA arrangement from craniodorsal to caudoventral. (Reproduced with permission from Hanna: The SPA arrangement of the branches of the upper trunk of the brachial plexus: a correction of a long-standing misconception and a new diagram of the brachial plexus. J Neurosurg 125:350–354, 2016. Copyright American Association of Neurological Surgeons.) DS, dorsal scapular; Lt, long thoracic; UT, upper (superior) trunk; MT, middle trunk; LT, lower (inferior) trunk; SC, subclavius; S, suprascapular; PC, posterior cord; LC, lateral cord; MC, medial cord; U&L Ssc, upper and lower subscapular; TD, thoracodorsal; Ax, axillary; R, radial; LP, lateral pectoral; MCu, musculocutaneous; M, median; U, ulnar; MP, medial pectoral; MBC, medial brachial cutaneous; MABC, medial antebrachial cutaneous. 1 = phrenic nerve; 2 = upper trunk; S = suprascapular nerve; P = posterior division; A = anterior division; 3 = middle trunk; 4 = lower trunk; 5 = posterior cord; 6 = lateral cord; 7 = medial cord; 8 = axillary artery; 9 = upper and lower subscapular nerve; 10 = axillary nerve; 11 = radial nerve; 12 = musculocutaneous nerve; 13 = median nerve; 14 = ulnar nerve; 15 = medial antebrachial cutaneous nerve.

The human BP receives contributions from the ventral rami of the C5-T1 spinal nerves. It is further divided into three trunks: the upper (superior) trunk, mainly derived from C5 and C6; the middle trunk, from C7; and the lower (inferior) trunk, from C8 and T1. The trunks divide further into anterior and posterior divisions, which give rise to the lateral, medial, and posterior cords. The BP contains terminal branches that innervate the muscles and provide sensation to the upper limb. The upper trunk innervates the suprascapular, axillary, radial, musculocutaneous, and median nerves. The middle trunk innervates the axillary, radial, musculocutaneous, and median nerves. The lower trunk innervates the ulnar nerve, with contributions to the axillary, radial, and median nerves. Due to the vast network of connections between spinal nerves, it is challenging to determine the specific spinal level contribution to each nerve ( Fig. 1 ).

### Rat BP Gross Anatomy

**Fig. 2:**
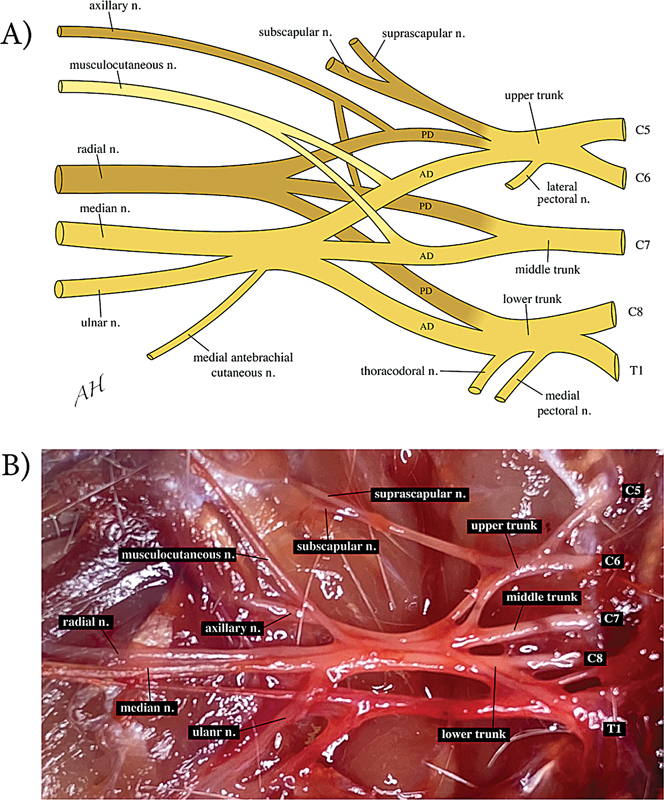
Sprague–Dawley rat brachial plexus. ( **A** ) Diagrammatic representation of the rat brachial plexus. The upper trunk is derived from C5 and C6; the middle trunk is derived from C7; the lower trunk is derived from C8 and T1. Each trunk splits into anterior divisions (AD) and posterior divisions (PD). ( **B** ) Photograph showing the anterior exposure of the SD rat's right brachial plexus with the clavicle removed. There is visible exposure of the C5-T1 spinal nerves, upper trunk, middle trunk, and lower trunk, which further divide into the following nerves: suprascapular, subscapular, axillary, musculocutaneous, radial, median, and ulnar. Note the formation of three trunks that split into two divisions each.

The rat BP also originates from the ventral rami of spinal nerves C5-T1. These five nerve roots come together to form three trunks. The upper trunk derives from C5 and C6, the middle trunk derives from C7, and the lower trunk derives from C8 and T1. Each trunk further divides into anterior and posterior divisions. The latter does not form clearly defined cords. The BP gives rise to terminal branches, which innervate specific muscles and provide sensory information to different regions of the upper limb. The cranial division of the upper trunk supplies the subscapular nerve and suprascapular nerves. The posterior divisions of the upper, middle, and lower trunks combine to form the radial nerve. The anterior divisions of the upper, middle, and lower trunk supply the median and ulnar nerves. The posterior divisions of the upper and middle trunk form the axillary nerve. The anterior divisions of the upper and middle trunk form the musculocutaneous nerve ( Fig. 2 ). There were some differences between the rats in terms of BP gross anatomy. In two specimens, T2 contributed to the ulnar nerve. Numerous interconnections existed between the upper, middle, and lower trunks, in two specimens, there was a clear merging of middle and lower trunks before branching.

### Histological Comparison

**Fig. 3:**
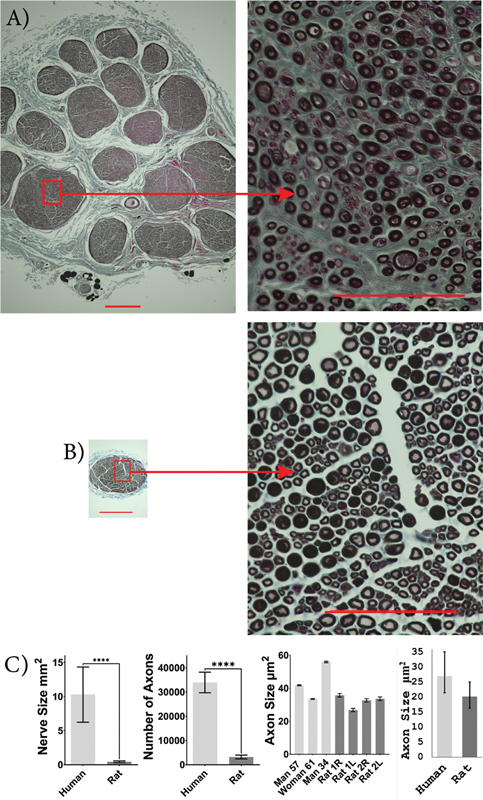
Human and rat median nerve characteristics. Images of osmium-fixed median nerves, counter-stained with Masson's trichrome for human ( **A** ) and SD rat ( **B** ). Scale bars: 400 µm on whole nerve images (left) and 100 µm on higher magnification images (right). Comparison between average human and rat median nerve size, number of axons present within the nerve, individual axon size, and sample population axon size ( **C** ). An unpaired, two-tailed Student's *t* -test was used to compare nerve size and axon count, and a mixed-effects ANOVA was used to compare axon size between humans and rats. The median nerve was larger in humans with more axons but there was no significant difference in axon size. Human, *n* = 3, and rat, *n* = 4. ^****^ *p* < 0.0001. Error bars indicate a 95% confidence interval.

Rat median nerves were significantly smaller in size compared to human median nerves (human: 10.29 [6.245 and 14.34] mm ^2^ ; rat: 0.44 [0.32 and 0.57] mm ^2^ ; *p* < 0.0001). The rat median nerves had significantly fewer myelinated axons than human median nerves (human: 33,958 [29,702 and 38,211]; rat: 3,189 [2,424 and 3,954]; *p* < 0.0001). The cross-sectional area of median axons was not significantly different between rats and humans (human: 27.4 [21.4 and 35.1] µm ^2^ ; rat: 20.2 [16.3 and 25.0] µm ^2^ ; *p* = 0.127; Fig. 3A–C ).

**Fig. 4:**
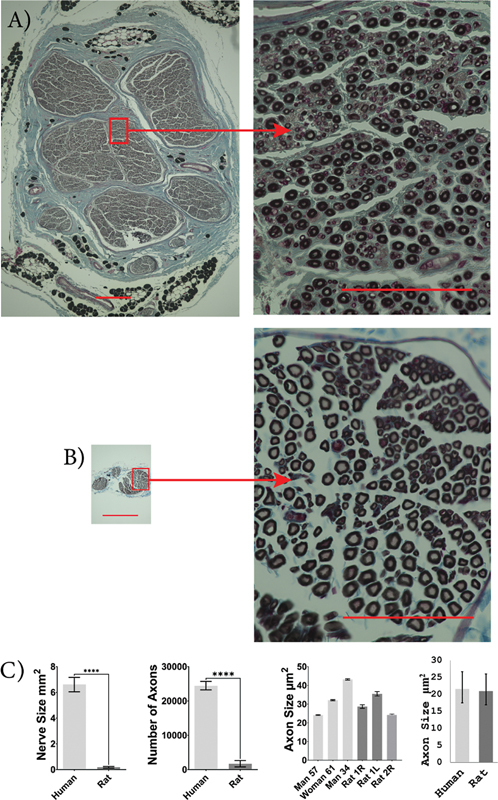
Human and rat ulnar nerve characteristics. Images of osmium-fixed ulnar nerves, counter-stained with Masson's trichrome for human ( **A** ) and SD rat ( **B** ). Scale bars: 400 µm on whole nerve images (left) and 100 µm on higher magnification images (right). Comparison between average human and rat nerve size, number of axons present within the nerve, individual axon size, and sample population axon size ( **C** ). An unpaired, two-tailed student's *t* -test was used to compare nerve size and axon count, and a mixed-effects ANOVA was used to compare axon size between humans and rats. The ulnar nerve was larger in humans with more axons but there was no significant difference in axon size. Human, *n* = 3, and rat, *n* = 3. ^****^ *p* < 0.0001. Error bars indicate a 95% confidence interval.

Rat ulnar nerves were significantly smaller in size compared to human ulnar nerves (human: 6.6 [6.0 and 7.2] mm ^2^ ; rat: 0.18 [0.11 and 0.25] mm ^2^ ; *p* < 0.0001). The rat ulnar nerves had significantly fewer myelinated axons than human ulnar nerves (human: 24,449 [23,224 and 25,675]; rat: 1,703 [760.9 and 2,644]; *p* < 0.0001). However, the cross-sectional area of ulnar axons did not significantly differ between rats and humans (human: 21.6 [17.5 and 26.7] µm ^2^ ; rat: 21.0 [16.9 and 26.0] µm ^2^ ; *p* = 0.853; Fig. 4A–C ).

**Fig. 5:**
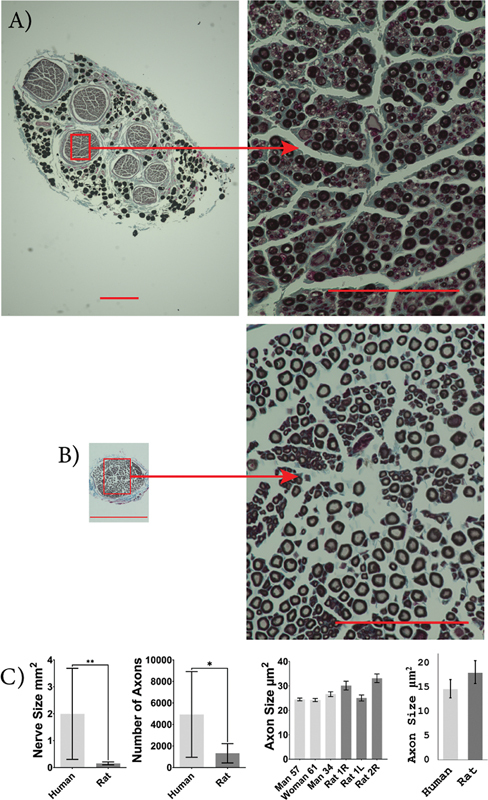
Human and rat musculocutaneous nerve characteristics. Images of osmium-fixed musculocutaneous nerves, counter-stained with Masson's trichrome for human ( **A** ) and SD rat ( **B** ). Scale bars: 400 µm on whole nerve images (left) and 100 µm on higher magnification images (right). Comparison between average human and rat nerve size, number of axons present within the nerve, individual axon size, and sample population axon size ( **C** ). An unpaired, two-tailed student's *t* -test was used to compare nerve size and axon count, and a mixed-effects ANOVA was used to compare axon size between humans and rats. The musculocutaneous nerve was larger in humans with more axons but there was no significant difference in axon size. Human, *n* = 3, and rat, *n* = 3. ^*^ *p* < 0.05, ^**^ *p* < 0.01. Error bars indicate a 95% confidence interval.

Rat musculocutaneous nerves were significantly smaller in size compared to human musculocutaneous nerves (human: 2.00 [0.30 and 3.69] mm ^2^ ; rat: 0.15 [0.099 and 0.21] mm ^2^ ; *p* = 0.0095). The rat musculocutaneous nerves had significantly fewer myelinated axons than human musculocutaneous nerves (human: 4,934 [950.5 and 8,918]; rat: 1,321 [429.6 and 2,213]; *p* = 0.019). The cross-sectional area of musculocutaneous axons was not significantly different between rats and humans (human: 14.6 [12.8 and 16.6] µm ^2^ ; rat: 18.0 [15.8 and 20.5] µm ^2^ ; *p* = 0.092; Fig. 5A–C ).

**Fig. 6:**
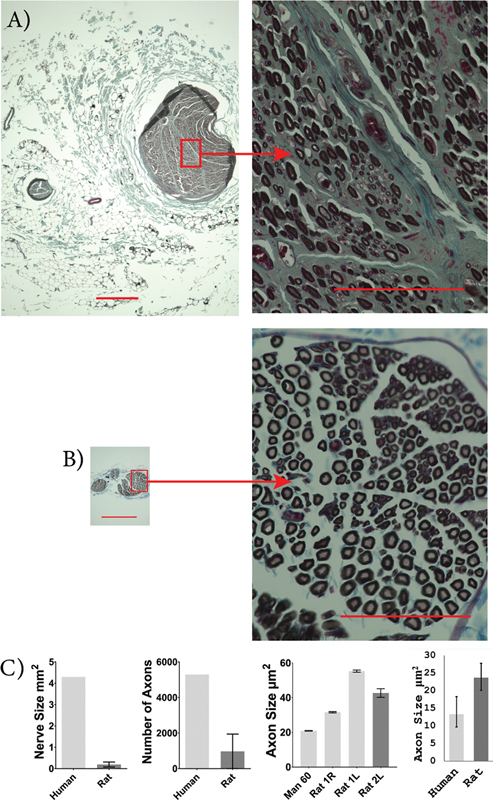
Human and rat suprascapular nerve characteristics. Images of osmium-fixed suprascapular nerves, counter-stained with Masson's trichrome for human ( **A** ) and SD rat ( **B** ). Scale bars: 400 µm on whole nerve images (left) and 100 µm on higher magnification images (right). Comparison between average human and rat nerve size, number of axons present within the nerve, individual axon size, and sample population axon size ( **C** ). A mixed-effects ANOVA was used to compare axon size between humans and rats (not significant). The suprascapular nerve was larger in humans with more axons but there was no significant difference in axon size. Human, *n* = 1, and rat, *n* = 3. Error bars indicate a 95% confidence interval.

**Fig. 7:**
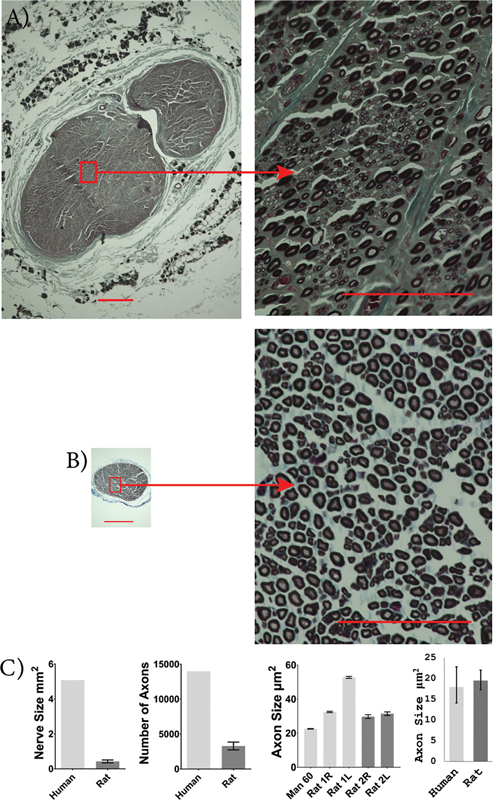
Human and rat radial nerve characteristics. Images of osmium-fixed radial nerves, counter-stained with Masson's trichrome for human ( **A** ) and SD rat ( **B** ). Scale bars: 400 µm on whole nerve images (left) and 100 µm on higher magnification images (right). Comparison between average human and rat nerve size, number of axons present within the nerve, individual axon size, and sample population axon size ( **C** ). A mixed-effects ANOVA was used to compare axon size between humans and rats (not significant). The radial nerve was larger in humans with more axons but there was no significant difference in axon size. Human, *n* = 1, and rat, *n* = 4. Error bars indicate a 95% confidence interval.

**Fig. 8:**
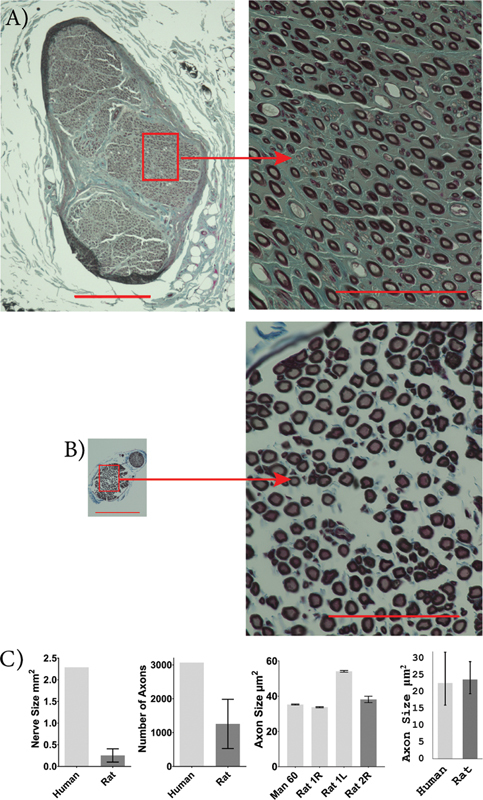
Human and rat axillary nerve characteristics. Images of osmium-fixed axillary nerves, counter-stained with Masson's trichrome for human ( **A** ) and SD rat ( **B** ). Scale bars: 400 µm on whole nerve images (left) and 100 µm on higher magnification images (right). Comparison between average human and rat nerve size, number of axons present within the nerve, individual axon size, and sample population axon size ( **C** ). A mixed-effects ANOVA was used to compare axon size between humans and rats (not significant). The axillary nerve was larger in humans with more axons but there was no significant difference in axon size. Human, *n* = 1, and rat, *n* = 3. Error bars indicate a 95% confidence interval.

Although only one human sample was available for the suprascapular, radial, and axillary nerve analyses, preventing any statistical analysis of nerve size and axon count, these nerves were smaller and contained fewer axons in rats compared to humans. Rat suprascapular nerves appeared smaller in size (human: 4.3 mm ^2^ ; rat: 0.19 [0.075 and 0.31] mm ^2^ ) and contained fewer myelinated axons (human: 5,291; rat: 968 [−6.681 and 1,943]) compared to the human suprascapular nerve. The cross-sectional area of the suprascapular nerve axons was not significantly different between rats and humans (human: 13.3 [9.7 and 18.3] µm ^2^ ; rat: 23.7 [20.1 and 27.8] µm ^2^ ; *p* = 0.055; Fig. 6A–C ). Rat radial nerves appeared smaller in size (human: 5.08 mm ^2^ ; rat: 0.43 [0.34 and 0.51] mm ^2^ ) and had fewer myelinated axons (human: 14,004; rat: 3,296 [2,733 and 3,858]) compared to the human radial nerve. Rat radial nerve axons were not significantly different from those in humans in terms of cross-sectional area (human: 17.9 [14.1 and 22.8] µm ^2^ ; rat: 19.5 [17.3 and 22.0] µm ^2^ ; *p* =0.586; Fig. 7A–C ). Rat axillary nerves also appeared much smaller in size (human: 2.29 mm ^2^ ; rat: 0.25 [0.10 and 0.41] mm ^2^ ) and had fewer myelinated axons (human: 3,068; rat: 1,256 [528.4 and 1,983]) compared to the human axillary nerve. Rat axillary nerve axons were not significantly different from those in humans in terms of cross-sectional area (human: 22.6 [16.0 and 31.8] µm ^2^ ; rat: 23.7 [19.4 and 29.0] µm ^2^ ; *p* = 0.834; Fig. 8A–C ).

## Discussion

The rat BP shares several anatomical similarities with humans, but differences in nerve arrangement and branch origins require careful consideration when comparing the two. Both species possess well-developed clavicles and share similar nerve arrangements, as the BP originates from spinal nerves C5-T1 and forms the upper, middle, and lower trunks. However, once the trunks divide, the rat BP does not regroup into cords as seen in humans but rather starts forming terminal branches. 19 Consequently, the collateral and terminal branches of the rat BP often have different origins compared to that of the human BP. In humans, the median nerve originates from the contributions of the lateral and medial cords. However, since the rat BP lacks clearly defined cords, the median nerve originates from the anterior division of the upper, middle, and lower trunks. The lack of a lateral cord in rats further impacts the origin of the musculocutaneous nerve, which is formed from separate branches of the anterior divisions of the upper and middle trunks. Another notable distinction is the presence of the posterior cord in humans, from which the axillary and radial nerves originate. 20 In rats, however, the posterior cord is absent and the axillary and radial nerves derive from separate dorsal branches with contributions from the upper, middle, and lower trunks. The rat radial nerve receives contributions from all three trunks, while the axillary nerve only receives contributions from the upper and middle trunks. These anatomical differences lead to variations in the origins of major nerves, complicating the development of translational therapeutic interventions for BPI.

Neuroanatomical and neurophysiological differences between rats and humans further complicate the direct translation of research findings from the rat model to clinical settings. The simpler organization of the rat's motor system limits its capacity for fine motor control and dexterous movements that are typical of humans. The behavioral outputs in rats, resulting from these simpler neural circuits, do not adequately replicate the complex motor behaviors observed in humans, leading to potential inefficacies and safety concerns in human applications. 21 These differences underscore the need for caution when using results from rat models to predict outcomes in human treatments.

The comparative histology results indicate no significant difference in axon size between SD rats and humans. While no significance was detected, the individual results of axon size showed the 34-year-old human male, with no underlying conditions, had, on average, larger axons than the developing rats and the aged humans. The lack of difference in axon size between the two species is likely attributable to the average age of the human sample, as 75% of the human samples utilized were over 50 years old and had underlying health conditions. Future studies should investigate this phenomenon with rats and humans of comparable biological age to deduce whether there is a significant difference in axon size between the two species. Although there was no significant difference in axon size, SD rats had significantly smaller nerves and significantly fewer myelinated axons in the median, ulnar, and musculocutaneous nerves compared to humans. No statistical analysis could be done for the suprascapular, radial, or axillary nerve sizes, but within the available specimens, human nerves were larger with more axons. Furthermore, the higher number of axons in the human nerves was likely due to the great demand for fine motor and sensory input associated with the human forelimbs' dexterous function.

This study contained some limitations. The first limitation was the range of underlying conditions and the ages of the humans used in this study. Due to the variability of human cadavers, it is difficult to acquire multiple fresh specimens that are similar in age, sex, and free of underlying conditions. The humans in this study had a variety of underlying conditions, while SD rats had no underlying conditions due to nutrition, weight, and overall health being monitored over the course of their lives. These underlying conditions may have impacted the quality of the human nerves sampled, as some of these conditions are associated with axonal demyelination, as seen in the case of Hepatitis C and its association with transverse myelitis. 22 Age is another factor that may have impacted the results of this study, as the ages of humans sampled ranged from 34 to 61 years old, while SD rats utilized had not yet reached full maturation. As animals age, axonal atrophy has been reported due to a decrease in the transport of cytoskeletal proteins in nerves. 23 Notably, the SD rats were approximately 101 days old and still undergoing skeletal growth, which does not slow until around 210 days. 24 This resulted in a comparison between fully developed humans and developing SD rats. Despite incongruencies between samples, valuable conclusions were still able to be drawn from nerve size and axon data.

Indeed, the SD rat is an accessible model with low husbandry cost and similar BP anatomy to that of the human. However, there are limitations when using SD rats as a model in BPI research. SD rats have high genetic variability, which poses challenges for replicating results. 14 Additionally, after nerve injury, axonal regeneration occurs at a faster rate in rats than in humans due to the rat's ability to regenerate tissues more rapidly and efficiently. 25 26 27 Appropriate awareness of differences in neuroregeneration is crucial when selecting suitable animal models.

While larger animals have been proven to be reliable and accurate models for BPI research, previous studies indicate that the SD rat model is suitable for generating preliminary data. However, not all conclusions derived from rat studies can be directly translatable to human trials. 14 16 28 Given the anatomical similarities and low husbandry costs, the SD rat model is justified for the initial phases of BPI research. Findings from rat studies should then be validated in larger animal models with closer physiological similarities to humans before advancing to clinical applications. Larger animals, such as the Wisconsin Miniature Swine, exhibit anatomical, physiological, and pathophysiological similarities to humans, making them an excellent model for furthering BPI research. 29 Similarities in the size of the BP in these larger animals allow for more complex therapeutic interventions to be researched.

## Conclusion

SD rats are an accessible and cost-effective model. Due to their overall anatomical similarities, rats are a great model for preliminary BPI research. However, differences in nerve size, branching patterns, and axon count exist between the rat and human BP. For complex therapeutic interventions, larger models with BPs more similar to humans should be considered.
